# Memory Performance Correlates of Hippocampal Subfield Volume in Mild Cognitive Impairment Subtype

**DOI:** 10.3389/fnbeh.2019.00259

**Published:** 2019-11-21

**Authors:** Kathryn M. Broadhouse, Loren Mowszowski, Shantel Duffy, Isabella Leung, Nathan Cross, Michael J. Valenzuela, Sharon L. Naismith

**Affiliations:** ^1^Sunshine Coast Mind and Neuroscience Thompson Institute, University of the Sunshine Coast, Sunshine Coast, QLD, Australia; ^2^Regenerative Neuroscience Group, Brain and Mind Centre, The University of Sydney, Sydney, NSW, Australia; ^3^Healthy Brain Aging Program, Brain and Mind Centre, The University of Sydney, Sydney, NSW, Australia; ^4^School of Psychology, Faculty of Science, The University of Sydney, Sydney, NSW, Australia; ^5^Charles Perkins Centre, The University of Sydney, Sydney, NSW, Australia; ^6^Sydney Medical School, The University of Sydney, Sydney, NSW, Australia

**Keywords:** mild cognitive impairment, hippocampus, subfields, memory, aging, neuroimaging

## Abstract

The increased understanding that neuropathology begins decades before symptom onset, has led to the conceptualization and widespread utilization of Mild Cognitive Impairment (MCI) as an important transitional state between healthy aging and dementia. Further subcategorization to MCI subtype has led to more distinct prognoses and it is widely considered that amnestic and non-amnestic MCI (aMCI, naMCI) likely have distinct pathophysiologies. Yet, accurately classification remains contentious. Here, we differentiate hippocampal subfield volume between subtypes, diagnosed according to stringent clinical consensus criteria, where aMCI is characterized based on deficits in delayed recall (rather than encoding). We then identify memory performance correlates to subfield volume and associations with long-term cognitive performance and outcome. 3D T1-weighted structural MRI was acquired in 142 participants recruited from the *Healthy Brain Aging (HBA) Clinic* and diagnosed with aMCI (*n* = 38), naMCI (*n* = 84) or subjective memory complaints (SMC; *n* = 20). T1-weighted datasets were processed with the cortical and hippocampal subfield processing streams in FreeSurfer (v6.0). Subfield volumes, and associations with baseline and longitudinal objective memory scores were then examined. Subfield volumes were found to differentiate clinical profiles: subiculum, CA1, CA4 and dentate gyrus volumes were significantly reduced in aMCI compared to both naMCI and SMC. CA1 subfield volume was shown to predict concurrent memory performance in aMCI, while dentate gyrus volume significantly predicted longitudinal verbal learning and memory decline in the entire cohort. Our findings demonstrate that using a more stringent diagnostic approach to characterizing aMCI is well justified, as delayed recall deficits are strongly linked to underlying volumetric subfield reductions in CA1, CA4 and the dentate gyrus, subfields known to be associated with mnemonic processes. Further research is now warranted to replicate these findings in other MCI samples.

## Introduction

Mild Cognitive Impairment (MCI), the transitional state between normal aging and dementia, is an important diagnostic entity in both clinical and research settings. Recent studies indicate that conversion rates to Alzheimer’s Disease (AD) are as high as 15% over a 2 year period (Petersen et al., 2018) and almost 45% over 5 years (Gauthier et al., 2006; Duffy et al., 2014). Further subcategorization into the clinical phenotypes of amnestic and non-amnestic MCI (aMCI and naMCI) (i.e., depending on whether impairment is evident in memory or non-memory cognitive domains, respectively) has shown that aMCI is associated with the most pronounced risk of conversion to AD; approximately 50% within 5 years (Gauthier et al., 2006; Petersen et al., 2009). For those with naMCI, the disease trajectory is less well defined (Jungwirth et al., 2012), but appears to be linked to higher risk and more frequent conversion to other dementia types (e.g., vascular dementia) compared to those whom are cognitively intact (Csukly et al., 2016).

The aMCI and naMCI subtypes are believed to be underpinned by different underlying pathophysiologies and disease trajectories. As such, to enable the most appropriate clinical feedback for patients (particularly where modifiable risk factors play a role), accurate characterization of the clinical subtyping of MCI and accompanying neurodegeneration is particularly important (Hughes et al., 2011; Norton et al., 2014). Another pertinent cohort when investigating cognition in aging are those that present with subjective memory complaints (SMC) but who do not demonstrate objective impairment on testing. A recent meta-analysis has shown that approximately 2.3 and 6.6% of SMC will progress to dementia and MCI per year, respectively (Mitchell et al., 2014). Subsequently, it has been suggested that SMCs may be a “pre-MCI” stage in the evolution of normal aging to clinical AD (Steinberg et al., 2013). By definition SMCs perform within a “normal range” on standard psychometric measures and although the individuals themselves, close family or friends, report subtle decline in cognitive abilities, they are otherwise still healthy community dwelling older adults. They are therefore health seeking and as such make an appropriate control group when investigating disease progression in clinic-based settings.

Pathophysiologically, MCI and the early stages of AD are characterized by increased neurodegeneration of the hippocampus and entorhinal cortex (Frisoni et al., 2010; Mu and Gage, 2011). Imaging studies have shown that hippocampal atrophy, specifically within the CA1 region and subiculum, not only correlates with cognitive impairment severity (Jack et al., 2000; Morra et al., 2009), but is associated with increased risk of conversion from MCI to AD (Apostolova et al., 2006; Costafreda et al., 2011). Although there is increased recognition that aMCI and naMCI are different phenotypes, relatively few studies have investigated the underlying cortical and hippocampal differences between them. Subfield analysis in healthy controls, MCI and AD patients has highlighted the presubiculum and subiculum as possible predictors of memory performance in MCI and suggests that prevalent atrophy of the presubicular-subicular complex is apparent from the early phases of AD (Carlesimo et al., 2015). Furthermore, significant reductions in whole hippocampal volume in aMCI relative to naMCI and controls has been shown (Csukly et al., 2016). However, a more in-depth subfield differentiation analysis, in particular with focus on association with objective memory performance has not been carried out in an early stage cohort or between MCI subtypes.

Regarding hippocampal-dependent memory deficits to categorize subtypes, we have observed that clinicians and researchers have utilized assessments of both new learning and delayed recall in characterizing aMCI (Bondi et al., 2014; Edmonds et al., 2015). However, our group has applied a more stringent criterion whereby only those demonstrating objective deficits *in delayed recall* are denoted as aMCI. Those with deficits in new learning, but not in delayed recall, or in other cognitive domains, are classified as naMCI. This deliberate methodological decision can be justified on both neuropsychological theoretical and evidence-based grounds. New learning is heavily dependent on sound and efficient encoding processes, which are frequently impacted by other cognitive skills including attention regulation and executive functions (e.g., with respect to strategic or effortful encoding strategies) that may in turn be linked to prefrontal cortex integrity. Delayed or episodic memory, however, is perhaps more specifically tied to hippocampal functioning and indeed delayed recall measures have been shown to be some of the most highly accurate predictors of progression to AD in both clinical and epidemiological samples (Gauthier et al., 2006). This more stringent approach to differentiating aMCI and naMCI may therefore be critical to ensuring more accurate diagnosis as well as exploring relationships with neuropathological markers of hippocampal functioning.

Accordingly, the aim of this study was to (a) determine entorhinal cortical and hippocampal subfield biomarkers that differentiate MCI subtypes and even those presenting with SMC; (b) identify whether our more stringent diagnostic approach is supported by whole hippocampal volumetric differences; and finally, (c) identify whether subfield volume is not only significantly associated with concurrent objective memory performance but also longitudinal memory decline.

## Materials and Methods

Participants were recruited from the *Healthy Brain Ageing (HBA) Clinic* at the Brain and Mind Centre, University of Sydney, Australia. The HBA clinic is a specialist early diagnosis and intervention research clinic that receives referrals from specialists and General Practitioners. Exclusion criteria for the clinic are: diagnosis of dementia or a Mini-Mental State Examination (MMSE) (Rovner and Folstein, 1987) score of less than 24, history of neurological illness, stroke or transient ischemic attack, head injury (with loss of consciousness >30 min), other medical conditions known to affect cognition, intellectual disability, insufficient English speaking skills for neuropsychological testing and history of prior or current substance abuse. In addition, for this study, we excluded any participant with current DSM-IV major depression. This research was approved by the Human Research Ethics Committee of the University of Sydney.

### Clinical Assessment

Using a semi-structured interview, a medical specialist (Neurologist, Psychiatrist or Geriatrician) recorded a full medical, clinical, psychiatric and medication history. For each participant, a Psychiatrist or research psychologist used the Mini International Neuropsychiatric Interview (van Vliet and de Beurs, 2007) to assess lifetime and current major depression. The 15-item Geriatric Depression Scale (GDS-15) (Greenberg, 2002) was also administered to measure depressive symptom severity. Clinician rated psychosocial functioning was assessed using the Social and Occupational Functioning Assessment Scale (SOFAS) (Rybarczyk, 2011) and severity of medical burden was measured using the Cumulative Illness Rating Scale – Geriatric Version (CIRS) (Linn et al., 1968) total score.

### Neuropsychological Assessment

The tests reported in this study formed part of a broader assessment battery (Duffy et al., 2014). This study focuses specifically on two tests of verbal learning and memory:

(i) The Logical Memory (LogMem) I and II subtests of the Wechsler Memory Scale (3rd edition) (Wechsler, 1997a) were used to measure encoding and recall of structured verbal material. Age-scaled scores (ASS) were computed according to normative data (Wechsler, 1997a).
(ii) The Rey Auditory Verbal Learning Test (RAVLT) (Lezak, 1982) word list task was used to assess unstructured verbal learning over five trials (RAVLT 1-5) and memory after a 20-min delay (RAVLT A7). Standardized *z*-scores were calculated using age and education corrected normative data (Senior, 1999).

For descriptive purposes, we also report MMSE (Folstein et al., 1975) scores and estimated premorbid intellectual functioning using the Wechsler Test of Adult Reading (Wechsler, 1997a).

### Group Diagnoses

Diagnosis was confirmed based on consensus rating of at least three clinicians. A clinical diagnosis of MCI was obtained using Winblad’s criteria (Winblad et al., 2004) where cognitive decline was defined as a deficit of at least 1.5 SDs from estimated premorbid functioning on objective neuropsychological tests, relative to age- and education-adjusted normative data. Each participant was required to have subjective and objective cognitive decline, but with the general preservation of function (i.e., only minimal change, if any, in basic and complex activities of daily living). Within the HBA clinic aMCI was defined specifically by impairment on measures of delayed recall [assessed with the Logical Memory II total score (Wechsler, 1997b); Rey Auditory Verbal Learning Test (RAVLT) trial 7 total score (Schmidt, 1996)]. In cases where only new learning (e.g., Logical Memory I total score or RAVLT 1-5) was impaired but delayed recall was intact, the individual was categorized as naMCI. SMC participants were identified as individuals who when recruited through HBA reported subtle decline in cognitive abilities on the Likert scale, yet upon neuropsychological testing, performed within the normal range. The five-point Likert scale identifies SMC by asking participants “*In general, how would you rate your memory?*.” Those rating their memory as ‘fair’ or ‘poor’ but with intact cognition were defined as SMC (Ganguli et al., 2004; Purser et al., 2006).

### MRI Acquisition and Analysis

A neuro-MRI protocol was acquired on a 3T GE Discovery MR750 Scanner (GE Medical Systems, Milwaukee, WI, United States), as described in Duffy et al. (2014). Entorhinal cortical thickness and hippocampal volumetric analyses were performed on a 3D T1-weighted, structural MRI sequence (1 mm isotropic resolution, matrix = 256 × 256, TR/TE/TI = 7.13/2.69/450 ms, flip angle = 12°) with the FreeSurfer(v6.0) cortical and hippocampal subfield processing stream (Reuter et al., 2010; Iglesias et al., 2015). All analysis was carried out on raw entorhinal cortical thickness measures (mm) and normalized subfield volumes and reported as percentage of intra-cranial volume (ICV). Whole-brain segmentations were visually inspected and manually adjusted where necessary before being processed through the subfield processing stream. Finally, subfield segmentations were then visually inspected to check for apparent errors in segmentation.

### Statistical Analysis

#### T1-Weighted Subfield Segmentation Validation

Currently, the “gold standard” hippocampal subfield segmentation streams utilize a dedicated T2-weighted, high in-plane spatial resolution (0.4 mm in plane) acquisition (HighResT2) (Iglesias et al., 2015). This is important in aging studies as extensive atrophy can distort hippocampal canonical morphological relationships and diminish gray-white matter contrast. Continuous recruitment to the HBA clinic meant that initial MRI protocols did not include this HighResT2 scan. We therefore sought to internally corroborate the T1-weighted subfield segmentations against a the HighResT2 output in a more recent HBA cohort. Bivariate correlation analysis was used to compare FreeSurfer-based 3D T1-weighted and HighResT2 volumes. T1-weighted subfield segmentations with a Pearson’s correlation of ≥0.9 with HighResT2 were deemed robust.

#### Group Analysis

Significant differences between age at MRI scan, years of education, and depressive symptomatology (GDS-15) was determined by a One-Way ANOVA. A Chi-Squared test was determined differences in female/male ratios across groups. A One-Way ANCOVA established statistically significant differences in normalized verbal learning and memory scores and cognitive performance between the clinical groups. Age, years of education, sex and GDS-15 were used as covariates. Finally, significant group differences in right and left entorhinal cortical thickness, whole hippocampal and subfield volumes were assessed using a One-Way ANCOVA controlling for covariates.

#### MCI Subtype Criteria Validation

The legitimacy of the stringent (i.e., requiring deficits in delayed recall for aMCI) HBA subtype classification (“*stringent criteria grouping*”) was investigated; naMCI participants demonstrating deficits in new learning were recoded to aMCI following the general (i.e.,- deficits in memory OR learning accepted as aMCI) criteria (“*general criteria grouping*”). A stepwise, multiple discriminant analysis was used to investigate if any of the predictor variables (subfield volumes and demographics) differentiated between SMC, aMCI and naMCI groups using the “*stringent criteria*” grouping and “*general criteria*” grouping.

#### Association to Cognitive Performance

Relationships between concurrent delayed recall performance scores and left whole hippocampal and the subfield volumes were investigated on a group basis with partial regressions controlling for age and education in both MCI subtypes separately. Fisher *r*-to-*z* transformation was used to investigate the significance of the difference between partial regression correlation coefficients.

#### Longitudinal Cognitive Assessment Analysis

For a subset of 75 participants, longitudinal cognitive assessment data was available. A repeated measures ANOVA assessed significant differences in decline in cognitive performance between groups between baseline and follow up assessment. A backward stepwise multiple linear regression (MLR) sought to identify if any baseline subfield volume measures significantly predicted follow-up verbal learning and memory performance. Covariates, time between assessment and subfield volumes were entered into the initial model.

All statistical analyses were carried out in SPSS (IBM, Armonk, NY, United States. Release 24). To correct for multiple comparisons a *post hoc* Bonferroni multiple comparisons between groups were carried out for all ANOVA and ANCOVA analyses and results presented are corrected *p*-values.

## Results

### Hippocampal Subfield Segmentation Corroboration

**FIGURE 1:**
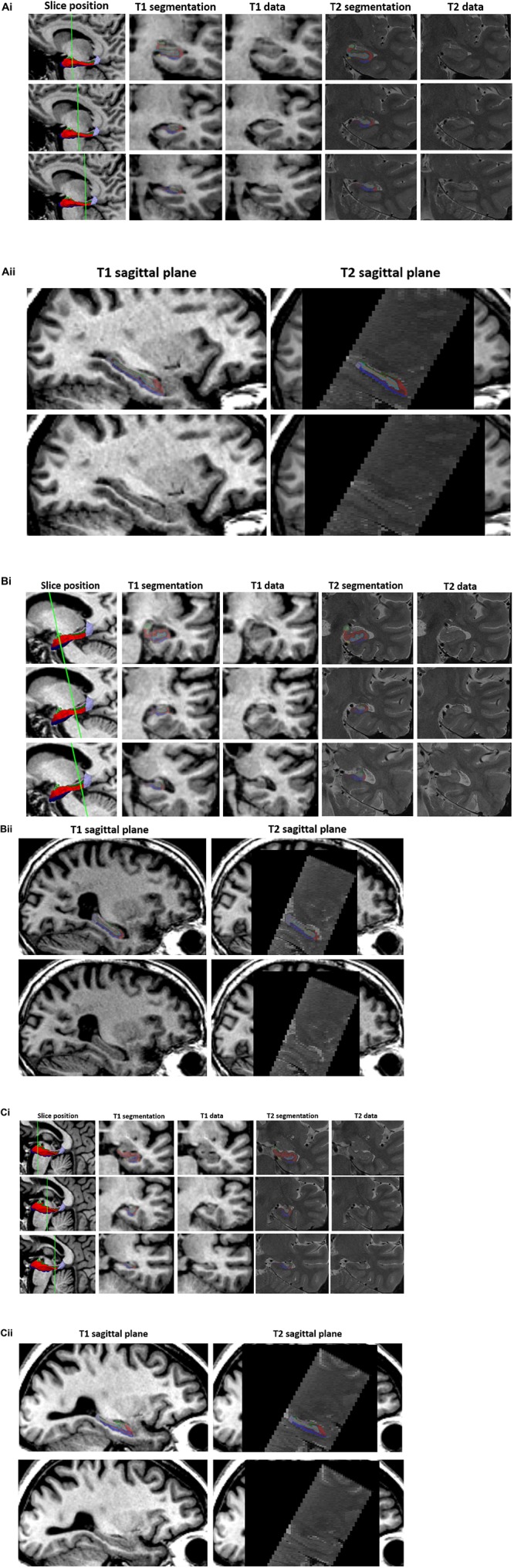
T1 vs. T2 segmentations. 3D T1-weighted and T2-weighted segmentation outputs are given for three example participants **(A–C)** in the validation subset. (i) Three slices are shown through the hippocampus (1st column) with T1 segmentation and raw T1 data (2nd and 3rd column) and T2 segmentation and T2 raw data (4th and 5th column) of the equivalent slice. T1-weighted datasets have been re-sliced to T2 slice orientation. The left hippocampal segmentation has been removed to show the underlying T1 (3rd column) and T2 (5th column – data shown in native T2 space) data. (ii) Sagittal T1 (left column) and T2 (right column) structural scans are shown with (top) and without (bottom) the respective hippocampal segmentations. Segmentations opacity has been reduced to visualize the corresponding structural scan underneath. Internal structures/boundaries can clearly be seen in both the T1 and T2 raw data and overlays displaying the corresponding segmentation indicate that these boundaries have been adequately followed in both the T1 and T2 based segmentation streams.

Segmentation corroboration was carried out in 79 participants recruited through HBA, diagnosed with SMC (*n* = 19), naMCI (*n* = 26), aMCI (*n* = 25) or AD (*n* = 8) [mean age = 69.0 years (SD = 8.4)]. Twenty-two of this cohort also had corresponding cognitive and MRI assessments, met diagnostic criteria and therefore their 3D T1-weighted data were also included in the principal analysis below. Five out of ten subfields met this criterion [subiculum, presubiculum, CA1, CA4 and dentate gyrus (Supplementary Figure 1)]. Accordingly, only these validated subfields were included in subsequent analyses. Examples of subfield delineation and the underlying 3D T1 and T2 -weighted data are given in Figure 1. These three examples were chosen at random from the MCI subjects and T1 datasets were re-sliced onto T2 to provide visual validity of segmentations.

### Sample Demographics and Neuropsychological Performance

Of the total sample (*n* = 142), 20 had SMC, 38 were diagnosed with aMCI and 84 with naMCI. There was no significant difference in age, years of education and GDS-15 scores between groups. However, as all are important risk factors for cognitive decline these were still controlled for as covariates. There was a significant association between female/male ratio and group (*χ*^2^ = 10.6, *p* = 0.005) with more females in the SMC group. Considering our *stringent criteria* for aMCI, unsurprisingly this group demonstrated significantly reduced learning and delayed recall as well as and greater global cognitive impairment (MMSE) when compared to SMC and naMCI groups. SMC and naMCI group did not differ statistically in terms of their memory performance. Results shown in Table 1.

**TABLE 1:** Sample demographics and cognitive performance: mean (SD) values for individual groups and total sample.

	**SMC**	**aMCI**	**naMCI**	**Total**	***F*/*X*^2^**	**Pairwise comparison**
Number	20	38	84	142	–	–
Sex (M/F)	4/16	23/15	30/54	57/85	10.6^∗∗∗†^	–
Education, years	13.5 (2.6)	14.3 (3.2)	13.5 (3.2)	13.7 (3.1)	0.8	–
Age at scan, years	67.6 (8.3)	67.6 (8.4)	66.5 (8.0)	66.9 (8.1)	0.3	–
GDS-15,/15	2.8 (2.4)	3.7 (3.0)	4.5 (3.9)	4.0 (3.5)	2.2	–
MMSE,/30	28.9 (0.4)^‡^	27.6 (0.3)^‡^	28.9 (0.2)^‡^		9.4^∗∗∗^	aMCI<naMCI<SMC
LogMem I ASS	12.2 (0.7)^‡^	7.5 (0.5)^‡^	10.3 (0.4)^‡^	−	15.2^∗∗∗^	aMCI<naMCI<SMC
LogMem II ASS	12.5 (0.7)^‡^	7.0 (0.5)^‡^	11.3 (0.3)^‡^	−	34.0^∗∗∗^	aMCI<naMCI<SMC
RAVLT 1-5, *z*-score	0.44 (0.2)^‡^	−1.25 (0.1)^‡^	−0.06 (0.1)^‡^	−	33.6^∗∗∗^	aMCI<naMCI<SMC
RAVLT A7, z-score	0.49(0.2)‡	−1.40 (0.1)^‡^	0.11 (0.1)^‡^	−	44.8^∗∗∗^	aMCI<naMCI<SMC

*^‡^Indicates marginal means and standard error. Sex, age, years of education and GDS used as covariates. ^†^Chi-squared. ^∗∗∗^*p* < 0.001. GDS = Geriatric Depression Scale, 15-item; MMSE = Mini Mental State Examination; LogMem I ASS = Logical Memory I age-scaled score; LogMem II ASS = Logical Memory II age-scaled score; RAVLT 1-5 *z*-score = standardized *z*-score of the Rey Auditory Verbal Learning Test total score over 1-5 verbal learning trials; RAVLT A7 *z*-score = Standardized *z*-score for the Rey Auditory Verbal Learning Test A7 delayed (20-min) memory recall.*

### Subfield Volumes Differentiate MCI Subtypes

**FIGURE 2:**
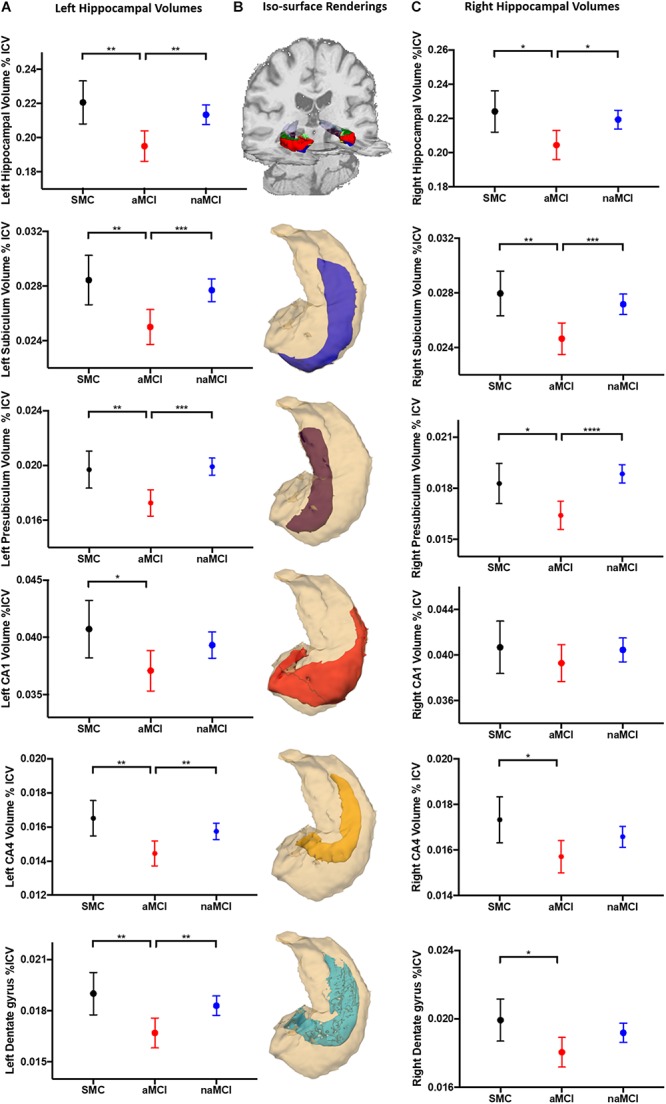
Hippocampal volumetric analysis. Marginal mean (SD) plots of left **(A)** and right **(C)** whole hippocampal, and the memory associated subfields subiculum, presubiculum, CA1, CA4 and dentate gyrus volumes from SMC, aMCI and naMCI patient groups. Significant differences between patient groups from One-Way ANCOVA analysis are displayed (^∗^) revealing that left hippocampal subfields are significantly atrophied in aMCI patients compared to SMC and naMCI groups. This pattern remains but is not as prevalent in the right hippocampus. Iso-surface renderings of an example whole hippocampal segmentation superimposed on coronal and axial planes of a structural 3D T1-weighted dataset **(B)** are displayed for reference purposes. Subsequent individual subfields of interest (blue = subiculum, magenta = presubiculum, red = CA1, yellow = CA4, cyan = dentate gyrus) shown in relation to the whole hippocampus (cream) are displayed below. Tests of between subject effects revealed that of the covariates, GDS-15 scores were significantly associated with left CA4 and dentate gyrus volume.

Both left and right whole hippocampal volumes were significantly reduced in aMCI compared to both SMC and naMCI (Left: *p* = 0.003, *p* = 0.002. Right: *p* = 0.022, *p* = 0.012, respectively). This pattern extended to the subfield analysis (Figure 2). All five analyzed subfields were significantly reduced in the aMCI compared to SMC groups and all but CA1 when compared to naMCI. This pattern was not as pronounced in the right, with only subiculum and presubiculum showing significant reductions when compared to naMCI. Cortical thickness analysis revealed that only the right entorhinal cortex was significantly reduced when comparing aMCI to SMC (*p* = 0.048). Full results given in Supplementary Table 1. As hippocampal differentiation was more prominent in the left and as entorhinal thickness did not differentiate between MCI subtype, all subsequent analyses were carried out with left subfield volumes only.

### Stringent Criteria for Subtype Classification Is Supported by Subfield Volume

Recoding participants who had new learning deficits (*n* = 18) to aMCI in the “*general criteria*” grouping scheme led to 66 participants being re-coded as naMCI, and 56 re-coded as aMCI (SMC; *n* = 20). Preliminary exploration of our data revealed a clear separation of participants who were consistently classified as aMCI and naMCI in both grouping schemes. Supplementary Figure 2 shows RAVLT A7 *z*-scores plotted against presubiculum volume; where aMCI typically presented with smaller volumes correlating to poorer verbal delayed recall performance. The recoded participants, however, were spread throughout both aMCI and naMCI clusters. A stepwise multiple discriminant analysis revealed presubiculum volume as the sole significant variable that discriminates subtype in both grouping schemes. The “*stringent criteria*” grouping classification led to an overall increase in correctly classified participants (67.6% compared to 57.0%). More specifically, “*stringent criteria*” grouping led to 76.8% more accurate classification of aMCI participants than random assignment (40.6% with “*general criteria*” grouping) (Table 2).

**TABLE 2:** Stepwise multiple discriminant analysis results from **(A)** “*stringent criteria*” grouping and **(B)** “*general criteria*” grouping. Both analyses returned presubiculum volume as the sole discriminant function coefficient.

**Prior probabilities for groups**	**Classification results**					
	**Prior%**	**Cases used**	**Predicted group membership%**			
			**SMC**	**aMCI**	**naMCI**	**Total**
**(A)**						
“***Stringent criteria***” **grouping**						
SMC	4.1	20	0	20.0	80.0	100
aMCI	26.8	38	0	47.4	52.6	100
naMCI	59.2	84	0	7.1	92.9	100
**(B)**						
“***General criteria***” **grouping**						
SMC	14.1	20	0	30.0	70.0	100
aMCI	39.4	56	0	55.4	44.6	100
naMCI	46.5	66	0	24.2	75.8	100

*A. 67.6% of original grouped cases correctly classified. B. 57.0% of original grouped cases correctly classified.*

### CA1 Subfield Volume Predicts Memory Performance in aMCI

Partial regression analyses revealed that there was a clear differentiation between the significant predictors of delayed recall performance in both MCI subgroups. Specifically, smaller CA1, CA4 and dentate gyrus volumes were associated with poorer memory performance in aMCI. However, only presubiculum volume was significantly correlated with delayed recall performance (RAVLT A7 *z*-score) in the naMCI group (Table 3). The Fisher *r*-to-*z* transformation revealed that the association between CA1 and delayed recall performance (LogMem II ASS) was significantly larger in aMCI than naMCI (*z* = 2.13, *p* = 0.03) groups.

**TABLE 3:** Partial correlation results showing association between verbal memory scores and subfield volume correcting for years of education and age.

**Partial correlations**	**aMCI**						**naMCI**				
		**CA1**	**Subiculum**	**Presub**	**DG**	**CA4**	**CA1**	**Subiculum**	**Presub**	**DG**	**CA4**
LogMem II ASS	*R*^2^	0.40	0.25	0.26	0.35	0.33	−0.01	0.02	0.01	0.07	0.10
	Sig	**0.016**	0.146	0.130	**0.039**	0.050	0.945	0.883	0.90	0.543	0.373
RAVLT A7 *z*-score	*R*^2^	0.45	0.24	0.13	0.38	0.37	0.14	0.23	0.40	0.20	0.19
	Sig	**0.007**	0.156	0.443	**0.023**	**0.026**	0.199	0.035	**<0.001**	0.074	0.090

*LogMem II ASS = Logical Memory II age-scaled score; RAVLT A7 *z*-score = Standardized *z*-score for The Rey Auditory Verbal Learning Test A7 delayed (20-min) memory recall; presub = presubiculum hippocampal subfield; DG = dentate gyrus. Significant *p* values are indicated in bold.*

### Dentate Gyrus Volume Predicts Longitudinal Auditory Learning and Memory Decline

Seventy-five participants (SMC = 15, aMCI = 14 and naMCI = 46) had a follow-up cognitive assessment at mean = 2.9 years (SD 1.3). There was no significant difference in follow-up time between groups. Participants diagnosed with aMCI at baseline had significant decline in global cognition, verbal learning and delayed recall performance at follow-up assessment compared to SMC and naMCI (MMSE: *p* = 0.039, *p* = 0.030, LogMem I ASS: *p* < 0.0001, *p* = 0.001, LogMem II ASS: *p* < 0.0001, *p* < 0.0001 and RAVLT 1-5 *z-*score: *p* < 0.0001, *p* = 0.018, RAVLT A7 *z-*score: *p* < 0.0001, *p* < 0.0001, respectively). Participants diagnosed with naMCI also had significant decline in RAVLT 1-5 *z*-scores compared to SMC (*p* = 0.028). Full results are given in Supplementary Table 2. Diagnoses at follow-up assessment (Supplementary Table 3) revealed that 11 and 7% of the aMCI and naMCI participants remained in the same diagnostic group, while the rest converted subtype, progressed to dementia or reverted to a “no MCI” diagnosis. A backward stepwise MLR revealed that only dentate gyrus volume and age or years of education (where years of education were protective) were significant predictors of decline; smaller baseline dentate gyrus volume predicted decline in follow-up RAVLT verbal learning and delayed recall performance scores (Table 4).

**TABLE 4:** Backward stepwise multiple linear regression result showing resultant significant predictors of follow-up verbal learning and delayed recall.

	**Predictor variables**	**Overall *R*^2^**	**Overall model significance**
FU LogMem I ASS	Dentate gyrus: (*p* = 0.061, *R*^2^ = 0.05)	0.07	0.031
	Education: (*p* = 0.064, *R*^2^ = 0.05)		
FU LogMem II ASS	Dentate gyrus: (β = 0.25, ***p* = 0.025**, *R*^2^ = 0.05)	0.11	0.006
	Age: (β = −0.23, *p* = 0.045, *R*^2^ = 0.07)		
FU RAVLT 1-5 z-score	Dentate Gyrus: (β = 0.26, ***p* = 0.022**, *R*^2^ = 0.07)	0.15	0.001
	Time between assessments: (β = 0.26, *p* = 0.024, *R*^2^ = 0.069)		
FU RAVLT A7 z-score	Dentate Gyrus: (β = 0.27, ***p* = 0.020**, *R*^2^ = 0.71)	0.06	0.020

*Overall model significance and correlation are shown as well as individual predictor partial variation. Standardized beta values are shown for significant predictors only. FU LogMem I ASS = Logical Memory I age-scaled score at follow up; FU LogMem II ASS = Logical Memory II age-scaled score at follow up; FU RAVLT 1-5 total *z*-score = standardized *z*-score of the Rey Auditory Verbal Learning Test total score over 1-5 verbal learning trials at follow up; FU RAVLT A7 *z*-score = standardized *z*-score for the Rey Auditory Verbal Learning Test A7 delayed (20-min) memory recall score at follow up. Significant *p* values are indicated in bold.*

## Discussion

This study provides further insight into the distinct structural and more specifically hippocampal pathophysiologies that likely underpin memory decline in SMC and MCI subgroups. Consistent with previous studies (Winkler et al., 2014; Csukly et al., 2016) our findings show significant reduction of both right and left whole hippocampal volume in aMCI compared to naMCI and SMC. However, our work extends these previous findings, indicating that specifically focusing on the *delayed recall* characteristics of memory decline for defining MCI subtypes is not merely theoretical, but is supported by key neuroanatomical differences in subiculum and dentate gyrus subfields. These subfields are heavily implicated in mnemonic consolidation processes (O’Mara et al., 2000) and localized atrophy of these subfields are associated with risk of progression from MCI to AD (Apostolova et al., 2006, 2010).

Significantly, we have shown that hippocampal subfields in MCI are distinctly associated with verbal memory performance. Specifically, in those with aMCI, the dentate gyrus, CA1 and CA4 were associated with delayed memory recall performance. By contrast, in naMCI, the presubiculum was associated with delayed performance. Moreover, the association between CA1 and delayed recall performance was significantly stronger in aMCI than naMCI. This correlation was still present, but weaker (*R*^2^ = 0.34) when following the “*general criteria*” grouping (results not included). These differential relationships may reflect the varied etiological mechanisms underpinning MCI subtypes. In addition, a recent study (Li et al., 2016) found significant atrophy in left subiculum and presubiculum subfields in a subcortical vascular MCI group (a prodromal stage of vascular dementia) compared to healthy controls. This coupled with our results, suggests that the presubiculum may be particularly sensitive to vascular pathologies, which may be more characteristic of naMCI.

Importantly, in this study, not only are the naMCI and SMC groups comparable on memory performance, but there were no measurable differences in whole hippocampal or subfield volume. Referring to Supplementary Figure 2, SMC participants did not form their own cluster in an extended upper quadrant along the linear trend line but fell within the boundaries of the naMCI participants. The lack of observable and measurable difference supports the notion that cognitive change in naMCI is due to more diverse brain pathology, that is less likely attributable to early changes in Amyloid beta (Aß). Alternatively, it is equally plausible that both groups may fall earlier in the cascade of dynamic biomarkers of AD, and may still exhibit preclinical AD, according to positron emission tomography (PET) and cerebrospinal fluid (CSF) biomarkers for AD (Coutinho et al., 2015; Dubois, 2018). Unfortunately, in this study, such measures necessary for future exploration were not available.

Various hippocampal subfields have been shown to play clear and distinct roles in mnemonic processes. Recent functional MRI studies in humans have found localized fMRI activity in the anterior CA2, CA3 and subiculum during learning, and in the CA1, and the posterior subiculum during retrieval of novel associations (Suthana et al., 2015). This idea of an encoding/retrieval gradient along the longitudinal axis of the hippocampus (Prince et al., 2005; Suthana et al., 2015) suggests that our results correlating the volume of retrieval-associated CA1 in aMCI may be functionally relevant. Furthermore, the earliest neurodegeneration seen in AD occurs in the entorhinal cortex and then progresses to the hippocampus. Future work should focus on combining both structural and functional data to investigate the association between not only hippocampal, but whole-brain atrophy patterns, corresponding functional signatures and cognitive performance, thus providing further insight into the underlying mechanisms differentiating MCI subtypes.

Improved understanding of the specific subfields that correlate with memory decline, will lead to more targeted treatments. Aerobic exercise has already been suggested as a possible promoter of hippocampal plasticity, with studies generally revealing attenuated whole hippocampal atrophy over the duration of intervention (Smith et al., 2010; ten Brinke et al., 2015). Furthermore, our recent work in an MCI sample found that hippocampal subfields susceptible to volume loss in AD were protected by progressive resistance training (Broadhouse et al., 2017 Abstract Supplement). Similarly, ongoing work in the field of cognitive training has demonstrated that these interventions can induce underlying neurophysiological alterations in healthy older adults and those with MCI (Belleville and Bherer, 2012; Suo et al., 2016).

Although AD sensitive subfields were significantly reduced in aMCI and these participants had statistically poorer performance at follow-up, subfield volume is clearly not the sole factor in determining outcome. It has, however, been postulated that the dentate gyrus plays a critical function in mediating processes such as recall of sequential information and short-term memory (Kesner, 2007). In this regard, our results showing larger dentate gyrus volume at baseline assessment was significantly associated with better follow-up cognitive and memory performance in all groups supports the notion that preserved dentate gyrus volume may be directly or indirectly associated with key neuroprotective factors implicated in preservation of memory function over time. It is in these early stage disease groups where modeling hippocampal volume trajectory preceding memory decline could be most relevant and informative. Further work incorporating both longitudinal MRI and cognitive assessment data is needed to fully understand the multifaceted, transient nature of MCI subtypes and SMC.

Several large-scale studies from the Alzheimer’s Disease Neuroimaging Initiative (ADNI) have highlighted the need for the refinement of MCI diagnostic criteria and have suggested that improved methods may yield gains in biomarker and clinical trial study findings because of improvements in sample compositions of “true positive” cases and removal of “false positive” cases (Bondi et al., 2014; Edmonds et al., 2015). From a clinical perspective, our results suggest that screening for MCI should always incorporate measures of delayed memory recall rather than simply relying on new learning or acquisition. Currently, gross screening tools do not incorporate such measures. Recent studies aiming to rate disease progression and score disease state have found that delayed recall is the earliest biomarker to be become abnormal during transition from health to disease (Jedynak et al., 2012). Our findings suggest that further exploration of clinically useful neuropsychological tools that may detect very subtle memory impairment may be worth pursuing, particularly if they are sensitive to the earliest forms of memory decline. For example, tasks that utilize short-term memory binding (Fernandez et al., 2018) or accelerated forgetting (Weston et al., 2018) may be worth examining in preclinical and MCI periods, and in relation to AD biomarkers.

### Limitations

Although the above reported results suggest a structural delineation between MCI subtypes there are several limitations to consider. Firstly, although we have limited our analyses to corroborated subfields to provide some confidence in our findings, it is important to note that although internally validated from high-field data, the FreeSurfer segmentations do not necessarily represent ground-truth in anatomy and must be interpreted with caution (Iglesias et al., 2015). FreeSurfer segmentations have not been validated against manual segmentation and without high-resolution T2 data there is a lack of internal information to guide the segmentation template and subfield labeling leading to uncertainty in subfield classification within the hippocampus. Due to the large user base, future studies validating FreeSurfer to manual segmentations would provide more confidence in T1 generated results. Though, as with all MR sequences there are tradeoffs. The improved gray/white matter contrast is achieved with a spin echo sequence – a gradient readout that is inherently longer and resultant contrast that provides less signal or “entropy” than the T1-weighted counterpart. Additionally, T2-weighted scans are much more affected by patient motion than the T1-weighted scans (Yushkevich et al., 2015). Furthermore, to achieve reasonable scan times the T2 sequence is not 3D and as a result has anisotropic resolution (typically 0.4 × 0.4 × 2 mm). These factors need to be considered when carrying out subfield segmentation. In a cohort where hippocampal atrophy occurs along the anterior-posterior axis (along the *through-plan*e direction where partial voluming effects will be greatest in the T2-weighted acquisitions) and speed to reduce motion artifacts is priority, the faster, 3D T1-weighted, isotropic resolution sequence may often be preferable. Future investigation of hippocampal subfields in MCI subtypes should implement a combined T1- T2- weighted segmentation method that takes full advantage of the isotropic T1 within the atlas-to-target registration and segmentation pipeline (Yushkevich et al., 2015). Furthermore, caution should be taken with any automated pipeline, T1- or T2-based, and investigators should always visually inspect their datasets for visible internal structures such as the leptomeningeal tissue in the vestigial hippocampal sulcus that is often used as a segmentation landmark and make it suitable for subfield processing pipelines. Future advancements in imaging acquisition, shorter scan time and improved contrast, will also provide further improvements to segmentation pipelines.

Secondly, we cannot claim the HBA criteria represents the optimal method; we propose that it represents an important step toward improved clinical classification. Although Supplementary Figure 2 provides a rudimentary visual inspection, this trend was apparent for all performance/subfield correlation plots (not included here). Deficits in delayed recall (aMCI) were linked with significant subfield atrophy, however, reduced subfield volume did not always lead to deficits in delayed recall (e.g., recoded participants). Although discriminant analysis indicated that “*stringent criteria*” grouping led to more participants accurately classified when considering hippocampal subfield volume alone, there is a clear need for further improvement. This lack of clear subgroup classification criteria for a subset of MCI subjects is most certainly due to the current modest understanding of disease etiology, and the poorly understood role of cognitive reserve in this population.

Finally, the lack of inclusion of a true “healthy control” group may limit our interpretation of results. However, by definition SMCs do not display objectively amnestic phenotypes. Although the individuals themselves report subtle decline in cognitive abilities, this is not detectable on conventional neuropsychological tests (which may lack sensitivity for detecting very subtle change) and the SMCs are otherwise still healthy community dwelling older adults. Recent evidence suggests that individuals with SMCs may be at increased risk of dementia and have a higher rate of progression to MCI than non-health seeking older adults (Schultz et al., 2015). Subsequently, it has been suggested that SMCs may represent a “pre-clinical” stage in the evolution of normal aging to clinical AD and might represent a potential target for intervention trials. Such individuals may indeed meet criteria for preclinical AD based on biomarker evidence (Gordon et al., 2018). As the field of AD has begun to increasingly focus on prevention, earlier detection and diagnosis of the disease has become paramount. Therefore, neurostructural comparison between SMCs and MCI subtypes may be more meaningful when investigating disease progression.

In summary, our data demonstrate that structural MRI can aid in the differentiation of MCI subtypes and reveals significant differences in whole hippocampal and subfield volumes between SMC, naMCI and aMCI which are in turn, strongly linked to verbal learning and memory performance. Of clinical significance, our findings further demonstrate that using a stringent diagnostic approach to characterizing aMCI based on predominant deficits in *delayed* recall (rather than merely encoding) is well justified, as such deficits are strongly and specifically linked to underlying volumetric subfield reductions in CA1, CA4 and the dentate gyrus.

## Data Availability Statement

The datasets generated for this study are available on request to the corresponding author.

## Ethics Statement

The studies involving human participants were reviewed and approved by the Human Research Ethics Committee of the University of Sydney. The patients/participants provided their written informed consent to participate in this study.

## Author Contributions

KB, LM, SD, MV, and SN contributed to the conception and design of the study. KB and IL performed the image preprocessing and statistical analysis. NC organized the database. KB wrote the first draft of the manuscript. All authors contributed to the manuscript revision, read, and approved the submitted version.

## Conflict of Interest

The authors declare that the research was conducted in the absence of any commercial or financial relationships that could be construed as a potential conflict of interest.
