# Supplementary material for: Memory Performance Correlates of Hippocampal Subfield Volume in Mild Cognitive Impairment Subtype
Source: Front Behav Neurosci. 2019 Nov 21;13:259. doi: 10.3389/fnbeh.2019.00259 (PMC6897308; doi:10.3389/fnbeh.2019.00259)
Supplement: Supplementary file 1 [file Data_Sheet_1.docx]

**SUPPLEMENTARY FIGURES**

**
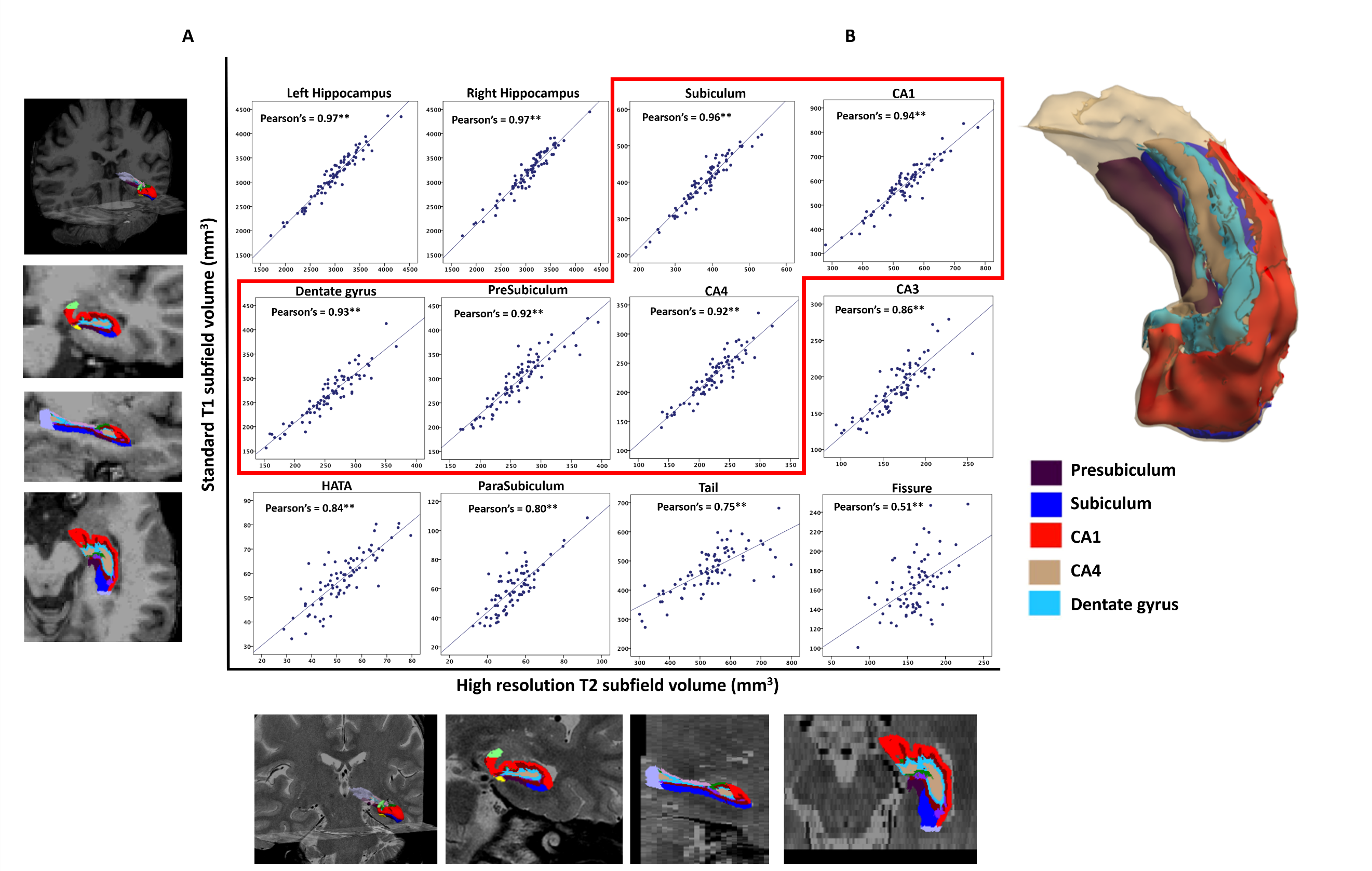
**

**Supplementary figure 1: High resolution imaging identifies hippocampal subfields accurately segmented using automated processing of standard T1-weighted MR images**

(**A**) Panel shows hippocampal subfield volumes determined by high resolution T2-imaging (acquired at 0.4mm in-plane resolution, slice thickness 2mm; x-axis) plotted against standard (1mm isotropic resolution; y-axis) T1 images processed with automated Freesurfer pipeline (v6.0). Each imaging protocol was run sequentially in each participant in the same scan session. Subfields with a cross-correlation coefficient > 0.9 were considered accurate at standard T1 1mm resolution. Those five subfields are highlighted in red box, and (**B**) depicted on an iso-surface rendering of the whole hippocampus. Only these five subfields are used in subsequent analyses. NB: dentate gyrus here refers to combined segmentation of granule cell layer, molecular layer and dentate gyrus.

**
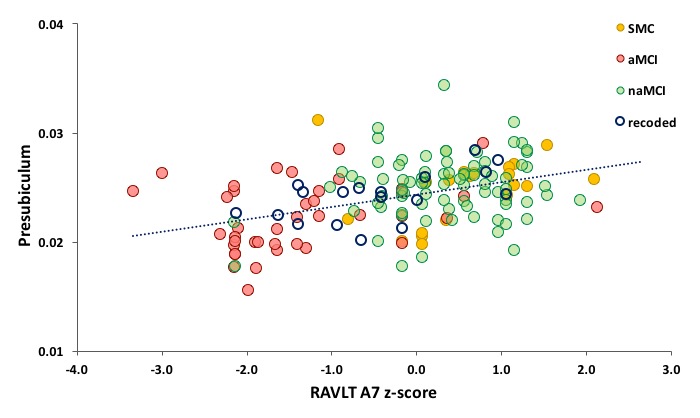
**

**Supplementary Figure 2: Presubiculum volume plotted against delayed recall performance.** Participants coloured by group: - consistently categorised as aMCI, naMCI and SMC in both LS and HBA grouping strategies and the 18 recoded subjects.

**SUPPLEMENTARY TABLES**

**Supplementary table 1: Cortical thickness analysis.** One-Way ANCOVA analysis of left and right entorhinal cortical thickness. Significant p-values are shown in bold.

| **Entorhinal thickness (mm)** |  |  | **Mean Diff** | **Std. Err** | **Sig.** | **95% Confidence Interval** | |
| --- | --- | --- | --- | --- | --- | --- | --- |
|  |  |  |  |  |  | **Lower Bound** | **Upper Bound** |
| **Left** | SMC | aMCI | 0.234 | 0.108 | 0.097 | -0.028 | 0.497 |
|  | SMC | naMCI | 0.133 | 0.096 | 0.506 | -0.1 | 0.365 |
|  | aMCI | naMCI | -0.102 | 0.077 | 0.564 | -0.288 | 0.085 |
| **Right** | SMC | aMCI | .250* | 0.102 | **0.048** | 0.002 | 0.498 |
|  | SMC | naMCI | 0.127 | 0.091 | 0.488 | -0.092 | 0.347 |
|  | aMCI | naMCI | -0.123 | 0.073 | 0.277 | -0.299 | 0.053 |
| Based on estimated marginal means | |  |  |  |  |  |  |
| * The mean difference is significant at the 0.05 level. | | | |  |  |  |  |
| b Adjustment for multiple comparisons: Bonferroni. | | | |  |  |  |  |

**Supplementary table 2: Follow-up analysis of decline with diagnosis.** Repeated measures ANOVA analysis of cognitive function, auditory learning and memory performance measures. Significant p-values are shown in bold.

|  |  |  | **Mean Diff** | **Std. Err** | **Sig.** | **95% Confidence Interval** | | |
| --- | --- | --- | --- | --- | --- | --- | --- | --- |
|  |  |  |  |  |  | **Lower** | **Upper** | |
| **MMSE** | SMC | aMCI | 1.191* | 0.466 | **0.039** | 0.046 | 2.335 | |
|  | SMC | naMCI | 0.158 | 0.380 | 1.000 | -0.775 | | 1.091 |
|  | aMCI | naMCI | -1.032 | 0.389 | **0.030** | -1.989 | -0.075 | |
| **LogMem ASS I** | SMC | aMCI | 4.819* | 1.066 | **0.000** | 2.206 | 7.432 | |
|  | SMC | naMCI | 1.544 | 0.853 | 0.223 | -0.546 | 3.635 | |
|  | aMCI | naMCI | -3.275 | 0.875 | **0.001** | -5.421 | -1.129 | |
| **LogMem ASS II** | SMC | aMCI | 6.126* | 0.990 | **0.000** | 3.699 | 8.554 | |
|  | SMC | naMCI | 1.244 | 0.792 | 0.362 | -0.698 | 3.186 | |
|  | aMCI | naMCI | -4.882 | 0.813 | **0.000** | -6.876 | -2.888 | |
| **RAVLT 1-5 z-score** | SMC | aMCI | 1.188* | 0.268 | **0.000** | 0.531 | 1.844 | |
|  | SMC | naMCI | .561* | 0.210 | **0.028** | 0.046 | 1.076 | |
|  | aMCI | naMCI | -0.627 | 0.222 | **0.018** | -1.171 | -0.083 | |
| **RAVLT A7 z-score** | SMC | aMCI | 1.575* | 0.293 | **0.000** | 0.856 | 2.294 | |
|  | SMC | naMCI | 0.512 | 0.230 | 0.088 | -0.052 | 1.076 | |
|  | aMCI | naMCI | -1.063 | 0.243 | **0.000** | -1.659 | -0.467 | |
|  | Based on observed means. | | |  |  |  |  | |
|  | The error term is Mean Square (Error) = .599. | | | |  |  |  | |
|  | * The mean difference is significant at the .05 level. | | | |  |  |  | |

**Supplementary table 3:** Group diagnoses at baseline and follow-up cognitive assessment. *3 aMCI and 1 naMCI had missing diagnosis information at follow-up. DBA = diagnosis at baseline assessment.

| **Group at baseline** | **DBA** | **no change** | | **converted to no MCI** | | | | **converted to aMCI** | | **converted to naMCI** | | | **progressed to dementia** | | |
| --- | --- | --- | --- | --- | --- | --- | --- | --- | --- | --- | --- | --- | --- | --- | --- |
|  |  | No. | % | | No. | % | No. | | % | | No. | % | | No. | **%** |
| **SMC** | 15 | 11 | 73.3% | | - | - | 2 | | 13.3% | | 2 | 13.3% | | 0 | 0% |
| **aMCI*** | 14 | 7 | 50.0% | | 2 | 14.3% | - | | - | | 1 | 7.2% | | 2 | 14.3% |
| **naMCI*** | 46 | 26 | 56.5% | | 13 | 28.2% | 8 | | 17.4% | | - | - | | 3 | 6.5% |
